# Effect of Panel Construction on the Ballistic Performance of Multiply 3D through-the-Thickness Angle-Interlock fabrIc Reinforced Composites

**DOI:** 10.3390/polym11020198

**Published:** 2019-01-24

**Authors:** Shengnan Min, Yuan Chai, Yanyan Chu, Xiaogang Chen

**Affiliations:** 1 Beijing Key Laboratory of Clothing Materials R & D and Assessment, Beijing Engineering Research Center of Textile Nanofiber, School of Materials Science and Engineering, Beijing Institute of Fashion Technology, Beijing 100029, China; 2 Henry Moseley X-Ray Imaging Facility, University of Manchester, Manchester M13 9PL, UK; yuan.chai@posrtgrad.manchester.ac.uk; 3 College of Textile Engineering, Zhongyuan Institute of Technology, Zhengzhou 450000, China; yychu@126.com; 4 School of Materials, University of Manchester, Manchester M13 9PL, UK; xiaogang.chen@manchester.ac.uk

**Keywords:** textile composites, ballistic performance, X-ray CT, 3D woven fabric, delamination

## Abstract

This paper studied the ballistic performance of 3D woven angle-interlock fabric reinforced composites with different types of panel construction. Two types of composites P10B and P17C were designed to have the same areal density of around 12 kg/m^2^ although they both had different ply areal densities and consisted of different numbers of plies. Non-perforated ballistic impacts were conducted on the two types of panels under the same level of impact energy. Post-mortem examination on the non-perforated panels was conducted through the cross-sectional images, planar projected delamination and 3D damage volume extracted from the non-destructive tests. Three distinctive sections of damage were segmented from the non-perforated panels, each indicating different material failure modes upon impact. Under the same areal density, the coarser composite panel P10B with a larger ply areal density and fewer reinforcement plies would result in less damage. The damage volume of P10B is nearly one-third that of the P17C. The findings are instructive for the design of 3D woven fabric continuously reinforced composites with doubly-curved shapes.

## 1. Introduction

The high-performance fibre reinforced textile composites have applications in an increasingly wider range of areas, including impact protective equipment. Ballistic impacts on composites result in localised deformation at the impact location and the composite materials fracture to absorb this impact energy. As a result, damages in forms of fibre breakage, matrix cracks, delamination between plies and indentation are often found. 

Due to the limitations imposed by the thickness of the conventional 2D reinforcing fabrics and the unidirectionally-laid fibre tows, the multiply reinforcing construction must be adopted in the making of advanced composites. The resin bonded multiple fabric layers within a composite panel allow for the development of delamination damage. Boussu [1] proposed that delamination should be encouraged before a multiply composite panel starts to absorb energy through the damage and deformation of fibres. Babu et al. [2] studied the failure modes of glass/epoxy unidirectional laminates and found that delamination was the main failure mechanism for thick panels. However, a weak binding between fabric layers would result in excessive indentation upon impact [3]. It can be lethal for the persons protected by the panel. For example, the standard for ballistic resistance of body armour NIJ-STD-0101.06 requires that the ballistic body armours of all protective levels should limit their indentations to 44 mm and below. The Chinese standard for police ballistic resistance of body armour GA141-2010 also requires a smaller indentation of 25 mm. Efforts have been made by different researchers to encourage stronger interlaminar binding, restrict damage and enhance impact resistance through different methods, such as fabric surface modification, matrix toughening, stitching, z-pinning and 3D fabrication with Z-bindings. 

Struszczyk et al. [4] modified the ballistic fabric surface by plasma-assisted chemical vapour deposition, resulting in rougher fibre surfaces and enhanced ballistic resistance. WanHanif et al. [5] found that the fracture toughness and ballistic resistance of Twaron^®^/epoxy composites both improved when the matrix was toughened with the multi-walled carbon nanotube. Hosur et al. [6] studied the ballistic resistance of stitched and unstitched woven fabric carbon/epoxy composite laminates. They determined that the delamination damage was contained within the stitch grid, while the ballistic limit was higher for the unstitched panels. Kang and Lee [7] found that the stitched composites exhibited a significant increase in impact energy absorption capability under repeated impacts. At the optimal stitch density, the ballistic efficiency of the stitched composite panels showed an increase of 10% compared to the unstitched woven fabric laminates. These indicate that the bonding between reinforcement layers should be carefully controlled in order for the delamination damage to contribute to impact energy absorption. 

**Figure 1:**
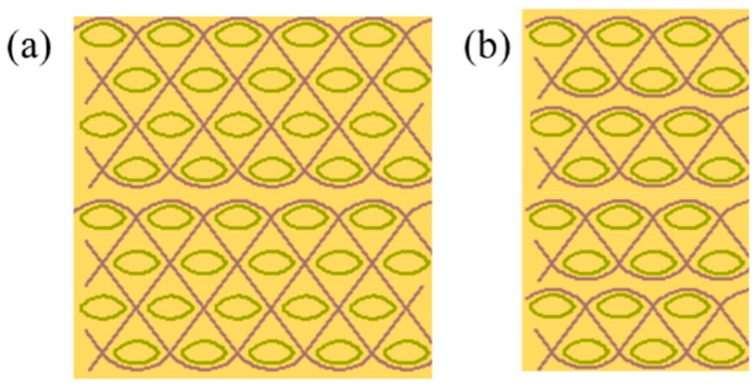
Multiply fabric reinforced composite panels: (**a**) 2-ply 3D woven 4-layer TTAI reinforced composite; and (**b**) 4-ply 2D plain weave fabric reinforced composite.

When designing and constructing multiply textile reinforced composite panels, the number of fabric plies used is a crucial factor. The use of thin 3D woven structures as advanced composite reinforcements would reduce the number for fabric plies required to reach a certain panel thickness. Figure 1 shows a 2-ply 3D woven 4-layer TTAI (Through-the-Thickness Angle-Interlock Fabric) reinforced composite and a 4-ply 2D plain weave fabric reinforced composite, which possess an equivalent thickness. The TTAI is a type of solid 3D woven fabric structure [8], which demonstrates high tensile, compressive and flexural performance [9]. Conventionally, it is composed by two sets of yarns, the straight wefts and binding (or interlocking) warps, as illustrated in Figure 1a. The straight wefts have alternate positions in different layers, which leaves enough space for binding yarns that go from top layer to bottom in a smooth path to bind layers together. The TTAI fabrics are highly mouldable because of their small resistance to shear deformation [10]. Hence, it has the potential to reinforce doubly-curved shapes without requiring time- and labour-consuming cutting or patterning processes [11]. Previous research on the effect of reinforcement continuity also indicates that the use of continuous fabric reinforcements encourages better ballistic resistance [12,13].

Although intensive researches have been conducted on the damage mechanisms of the 3D woven fabric reinforced composite under low-velocity impacts or quasi-static loadings [14,15,16,17,18], their ballistic impact responses and failure mechanisms are still under investigation. Wu et al. investigated the delamination toughness and impact damage tolerance of graphite/epoxy laminates experimentally using the Hopkinson pressure bar. They created controllable impulsive loading with varying magnitudes and investigated the impact responses of the composites under different impact energies [19,20]. In order to understand the role of the Z-fibres in impact energy absorption, Ghosh and De [21] conducted computational investigation on the ballistic response of 3D orthogonal woven structures. They concluded that the 3D reinforcement helped in improving penetration and impact resistance through both energy absorption and structural engagement. However, the Z-fibres were prone to debonding at crown ends and failed to arrest shear damage. Bandura et al. [22] studied the ballistic performance of different reinforcement architectures, including 2D plain woven, thick-section 3D orthogonal and 3D angle-interlock fabrics based on the Kevlar^®^/polypropylene system. The 3D armours demonstrated a ballistic limit that was higher by 2.4%–7% compared to the 2D ones, with the 3D angle-interlock structure having the highest limit. Conventional thin TTAI fabrics could be produced on dobby looms. Compared to the thick-section one-piece 3D woven fabrics, the use of multiply conventional TTAI fabrics as reinforcements for ballistic composites still allows delamination damage to develop. However, the effect of panel construction, regarding the number of fabric plies used and the fineness of each ply, has been rarely reported in the literature. 

The aim of this paper is to study the effect of panel construction on the ballistic performance of multiply TTAI reinforced composites. Their energy absorption, through-the-thickness damage morphology, planar damage area and 3D damage volume after ballistic impacts were examined. Experimental methods, including non-perforation ballistic impacts, through transmission ultrasonic C-scan and X-ray CT (Computed Tomography), were employed. The presented research in this paper aims to provide a deeper understanding of the ballistic performance and damage mechanisms of the multiply 3D TTAI fabric reinforced composites of different panel construction, which will ultimately be used to guide the design of composites in personal protection.

## 2. Materials and Methods

### 2.1. 3D TTAI Fabric Reinforced Composites

The high-performance para-aramid yarns Twaron^®^ provided by Teijin^®^ were used as the composite reinforcements. Each Twaron^®^ yarn was composed of 1000 filaments. To investigate the effects of reinforcement construction on the ballistic performance, two types of the conventional TTAI fabrics with different ply areal densities were designed. The specifications of the designed fabric reinforcements are provided in Table 1. 

To reach a similar panel areal density of around 12 kg/m^2^, two types of composites reinforced by the 3D TTAI fabrics (P10B and P17C) were designed, which are described in Table 2. The differences in the areal density of the single TTAI fabric ply result in the variations in the number of fabric plies required to build the composite panels. To be more specific, a heavier fabric ply would mean that a smaller number of fabric plies would be used to build the composite. 

**Figure 2:**
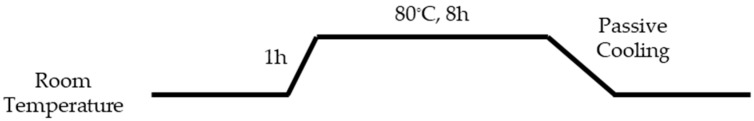
Curing program of Huntsman^®^ Araldite^®^ LY564 epoxy resin and hardener XB3486 system.

All the multiply fabric reinforced composites were produced through the vacuum-bag infusion method. The fabric plies were oriented along the same direction [0/0]_n_. The matrix used was a warm-curing epoxy system based on Araldite^®^ LY564 and a formulated amine hardener XB3486 from Huntsman^®^, which were mixed at a weight ratio of 100:34. The curing cycle for the infused panels is shown in Figure 2. The mechanical properties of the reinforcing fibre and cured resin matrix are listed in Table 3. The fibre volume fraction of the composites was evaluated by acid digestion. After this, the composite panels were trimmed into squares with the dimensions of 160 mm × 160 mm for ballistic impact tests and post-mortem analyses. 

### 2.2. Ballistic Performance Tests

**Figure 3:**
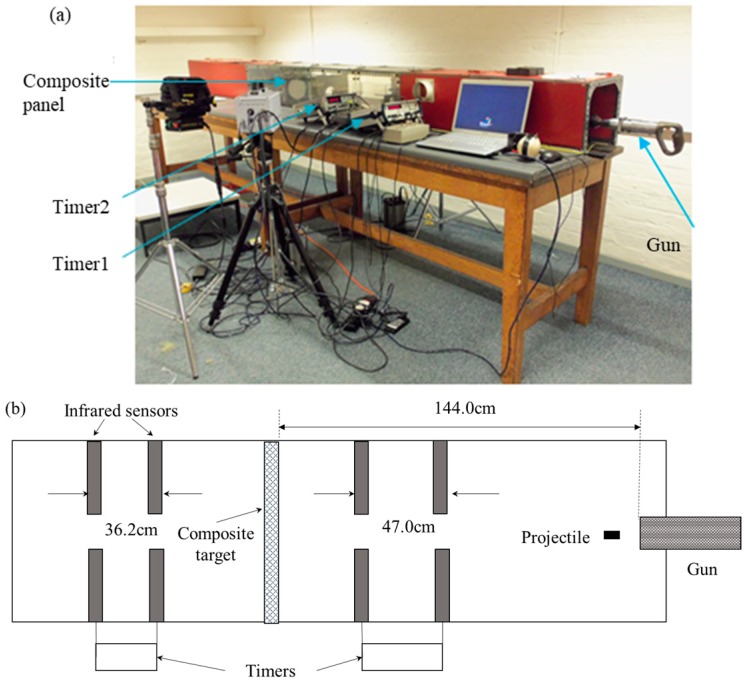
Setup of the ballistic impact: (**a**) Ballistic range; and (**b**) Schematic of the ballistic range for composite ballistic performance evaluation.

**Figure 4:**
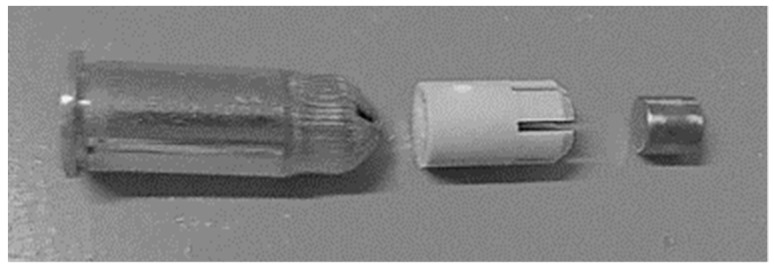
Projectile assembly of the ballistic impacts.

The ballistic performance of the composite panels was examined on a ballistic range, as illustrated in Figure 3. The amount of time it took for the projectile to travel through the two slots located before and after the target could be recorded by two timers each. The impact and residual velocities are calculated, given the distance travelled. For the non-perforated tests, only the impact velocities could be tracked and the residual velocities are zero because the projectile was stopped by the composite targets. The impact projectile is a steel cylinder of 1.06 g with a diameter and height of 5.5 mm. Propelled by a blank cartridge, the projectile impacts the targets at a velocity of 450–500 m/s. Details of the projectile assembly used for the ballistic impacts are provided in Figure 4.

The ballistic performance of the composite targets can be evaluated by the energy absorption (EA), which can be worked out based on Equation (1):

(1) $$EA=\frac{1}{2}m\left(V_{i}^{2}-V_{r}^{2}\right),$$

where *m* is the mass of the projectile in grams, *v_i_* and *v_r_* are the impact and residual velocities of the projectile, respectively. For non-perforated tests, the energy absorption *EA* is regarded as being equal to the impact kinetic energy carried by the projectile initially as the projectile residual velocity is zero. 

### 2.3. Non-Destructive Tests

After this, the ballistic impacted composites were subjected to non-destructive post-mortem examinations for their damage morphology. The planar damage areas, cross-sectional distribution and the 3D damage volumes were extracted by through transmission ultrasonic C-scan and X-ray computed tomography. 

The C-scan was conducted on a Midas NDT C-scan system with Zeus software used for image processing. The projections of damage from each reinforcement ply were superimposed into a planar damage distribution. The scan resolution is 72 × 72 pixels per inch^2^. The damage area can then be worked out by counting the number of pixels based on the grey scale differences. 

A Nikon Metrology320/225 kV custom bay (Henry Mosley X-ray Imaging Facility, University of Manchester, Manchester, UK) under a 225 kV X-ray source and a tungsten target was employed for the X-ray CT examination. Due to the differences in material composition between the steel projectile and the Twaron^®^/epoxy composite panels, the presence of steep edges in the projectile contour leads to dramatic changes in the number of X-rays transmitted and absorbed around the projectile. Hence, streaking and aliasing artefacts were noticed around the edges of the projectile [23]. To minimise such negative effects, a 1-mm Tin filter was applied while the contrast between composite and the background was also compromised. Details of the imaging parameters are listed in Table 4.

The composite impact damage, including fibre breakage, delamination and matrix cracks, can be determined based on the cross-sectional 2D image slices. These 2D image slices were combined and reconstructed into 3D volume files of the composites by Nikon Metrology software CT-Pro^®^. The 3D visualisation software Avizo^®^ was used for damage volume segmentation and analyses, which were conducted based on the greyscale thresholding technique. 

## 3. Results and Discussion

The composite panels P10B and P17C were subjected to ballistic tests and neither of them were penetrated by the projectiles. The two types of panels were found to be non-perforated under the impact velocities of 450–500 m/s (impact energy 101–125 J). The two panels that were impacted at an equivalent level of around 115 J were further investigated for their damage volume and damage distribution obtained from the tests using X-ray CT and C-scan. Details of the impact and the damage monitored are included in Table 5. 

### 3.1. Through-the-Thickness Cross-Sectional Damage Morphology at Impact Location

**Figure 5:**
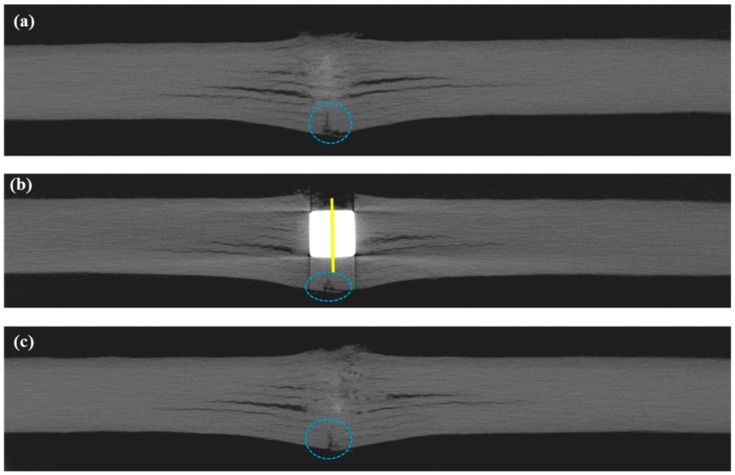
2D slice images around the impact location of panel P10B.

**Figure 6:**
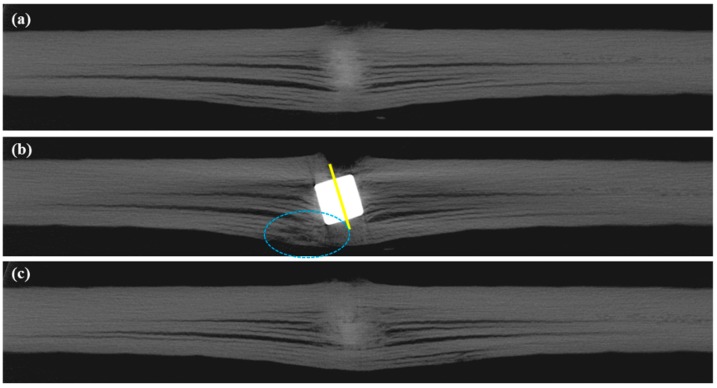
2D slice images around the impact location of panel P17C.

Although it is technically difficult to catch the movement of the projectile as it proceeds inside the composite panel, the 2D cross-sectional images obtained from X-ray CT provides an insight into the damages caused by the impact, which can be obtained in a non-destructive manner. Slice (b) presents the slice image through the centre of the projectile while slices (a) and (c) present the first and last slices without the projectile around the impact location. These slice images of composite plates P10B and P17C are illustrated in Figure 5 and Figure 6, respectively. Detailed post-mortem analyses were conducted on the projection images and the results are summarised in Table 6. 

The maximum thickness measures the thickness of the most indented position of the impacted panel. This value quantifies the depth of back-face indentation on the non-perforated panels. The penetration depth accounts for the distance from the impact face to where the projectile stops. To describe the severity of the damage induced, the penetrated percentage and thickness increase ratio are calculated. The penetrated percentage is the ratio of penetration depth to the maximum thickness of the panel. The thickness increase ratio is the maximum thickness increase to its thickness before impact. The increase in panel thickness at impact location is critical in determining the success of a ballistic panel, especially for their applications as body armours, since large indentations could also hurt the armour wearers. 

#### 3.1.1. Longitudinal View

We found that 12 plies out of 17 were penetrated in P17C, which also had more indents than P10B. The penetration depth accounts for 73.3% of the maximum panel thickness measured along the central axis of the projectile. The fracture damage in P10B was less severe, indicating a greater potential for resisting ballistic threats. The advantage of using fewer reinforcement plies with a heavier ply weight for constructing a multiply fabric reinforced ballistic composite was supported by these results. 

Based on the neat cuts on the entrance of the impacting projectile, it was inferred that the cylindrical projectiles impacted the panel perpendicularly. However, we noticed that the projectile stopped in P17C at an inclination angle of 12.64° compared to its original vertical position. Furthermore, there was also cracking of the reinforcing fabrics underneath the bottom left corner of the projectile. The cracking was highlighted by the dotted circles on Figure 6b. It could be assumed that the projectile proceeded further inside the panel before it was stopped by the last five fabric plies and travelled backwards until it reached its current position. The resistance from the rear plies of the panel slowed down the projectile before the kinetic energy of projectile was fully dissipated and rested on its final position. Due to the cracks highlighted underneath the left corner of the projectile in Figure 6b, the resisting force towards the projectile became weaker than that on the right-hand side. The crack on the last fabric ply also implies a tensile failure mode for the fibres in it. 

#### 3.1.2. Transverse View

Three distinctive regions of the deformation through the thickness of the composite panels were witnessed on both panels at the impact location. The widest-spread delamination always occurred at the particular reinforcement ply that the projectile stopped at. It is denoted as the last penetrated reinforcement ply. Section A was located on the first few plies near the entrance of the impacting projectile. The reinforcing fibres within the plies of Section A were found to be neatly cut by the projectile. There was no obvious deformation of fabric plies out of their original plane, except for the very first ply. The fibres are mainly broken due to transverse shearing. The shear plug cut out from Section A was compressed into Section B. The fibres in Section B were found to be severely stretched and deformed out of their initial ply planes, leading to fibre breakage and delamination. The reinforcing fabrics were not penetrated by the projectile in Section C as the projectile stopped at the exit of Section B. The cracking and bulging of the reinforcement plies were also noticed underneath the projectile. The rear plies of the panel may even break as witnessed in Figure 6b due to the stretching force from indenting. This partition scheme will help with the understanding of impact damage mechanisms and the design of multiply fabric reinforced hybrid ballistic composites. However, further studies need to be conducted in order to understand the distribution mechanisms of these three through-the-thickness sections.

### 3.2. Planar Damage Distribution

**Figure 7:**
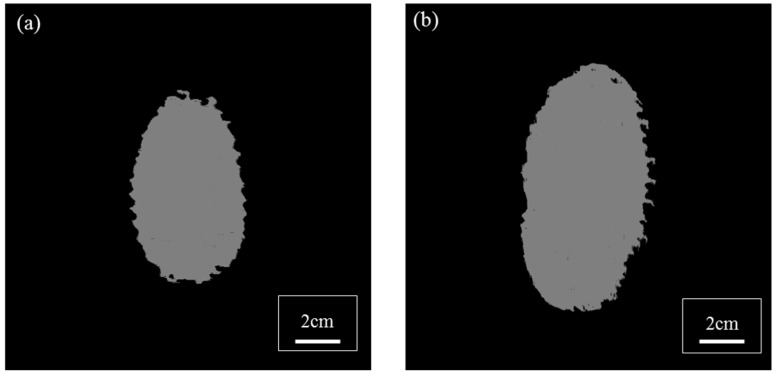
Planar damage areas of TTAI reinforced composites: (**a**) P10B; and (**b**) P17C.

Figure 7 outlines the planar projected damage extracted from the ultrasonic C-scan detection. This contour of the planar damage is elliptical in both panels. All of the reinforcing fabrics were arranged in an aligned orientation [0/0]_n_ and the long axes of the ellipses were found along the warp direction of the reinforcing TTAI fabrics. The lengths of the maximum lengths of the long and short axis of the three damage areas are listed in Table 7. 

We noticed that the damaged length in P17C along its warp direction is nearly double the size of that along its weft direction. The differences in the shapes of damaged areas is caused by the differentiated crimp levels along the warp and weft directions of the fabrics. It is understood that the warp direction of the conventional TTAI fabrics, such as B and C, has a smaller Young’s modulus and larger failure strain when compared to that of the weft direction. The mechanical properties of a single-ply conventional TTAI reinforced composite P1C are listed in Table 8. This difference is due to the higher crimp level of the binding warps compared to that of the straight weft yarns in the conventional TTAI fabrics. This suggests the need for an enhancement along the warp direction of convention TTAI fabrics for future ballistic panel design.

Under the similar ballistic threat level, the planar projected damage area of P17C is larger, as shown in Table 5. This also agrees with the cross-sectional delamination witness in Figure 5 and Figure 6. Thus, when the same amount of impact energy is absorbed, larger areas of materials were engaged in energy absorption through interlaminar failure. However, the through-the-thickness damage distribution in the panel cannot be reflected by these projected planar damages.

### 3.3. 3D Damage Volume

The use of 3D damage volume is an effective way of quantifying the severity of the delamination in the composite panel after ballistic impacts [12]. The 3D damage volume of the non-perforated composite panels was segmented, as listed in Table 5. The damage volume of P10B is nearly one-third that of the P17C. This means that given an equivalent amount of the impact energy being absorbed, more of the energy was dissipated through delamination of the reinforcing fabric plies for P17C than P10B. 

Compared to the other panel, P17B is a finer one with more fabric reinforcement plies and consequently, has a greater potential for delamination to develop. However, the level of delamination should be carefully controlled because it is closely related to the indentation depth of the composite panel after impact. As witnessed in this case from the cross-sectional damage morphology (Table 6), a higher thickness increase ratio was found in P17B than that in P10C.

**Figure 8:**
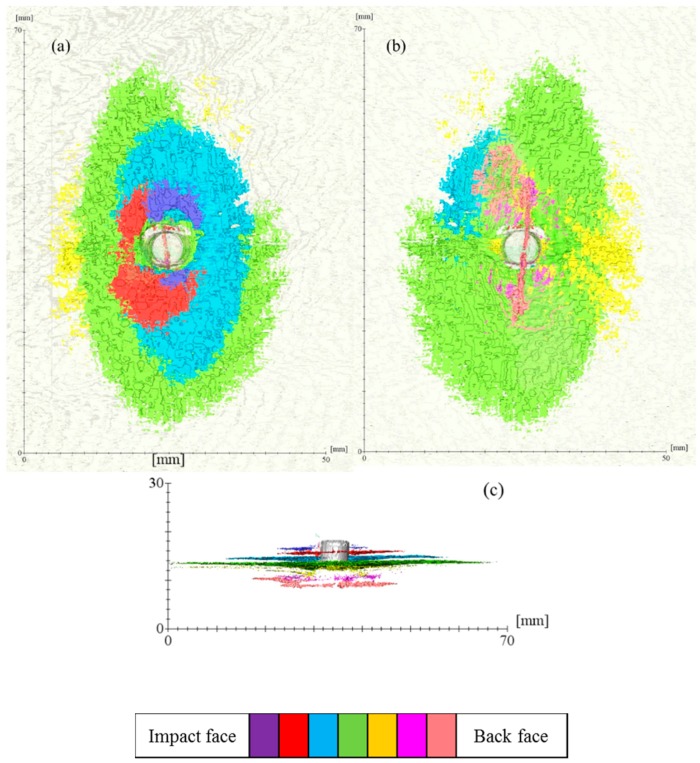
Delamination volume of P10B at different positions: (**a**) View from impact face; (**b**) View from back face; and (**c**) Cross-sectional view.

**Figure 9:**
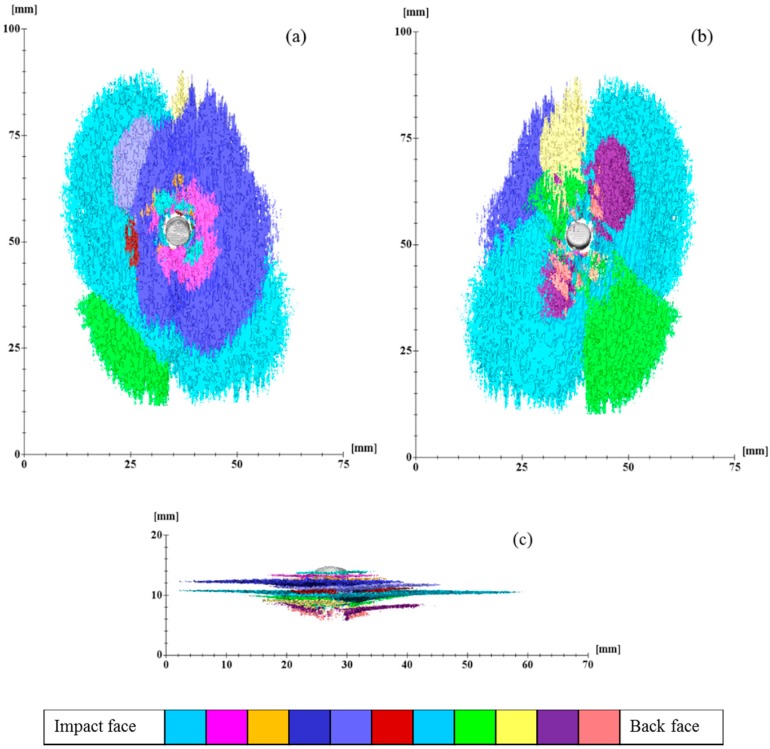
Delamination volume of P17C at different positions: (**a**) View from impact face; (**b**) View from back face; and (**c**) Cross-sectional view.

**Figure 10:**
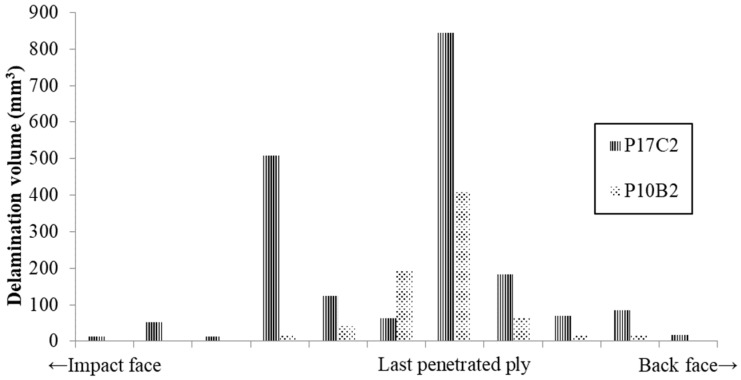
Distribution of delamination volume in each ply throughout panel thickness.

The delamination damage between each reinforcement ply was also segmented, as presented in Figure 8 and Figure 9, respectively. The colour codes in each figure represent the delamination volume in different delaminated plies. The sequence of the colour code is arranged according to the delamination location from the impact face towards the back face, which is indicated in the bar chart under each figure. The projected 3D damage volume was consistent with the planar views obtained from ultrasonic C-scan, as listed in Table 5. However, the 3D volume reflects the degree of damage more precisely throughout the panel thickness. The damage volume for each delaminated ply is summarised in Figure 10. Due to the differences in the total number of reinforcement plies in the three panels, we compared the delaminated plies that were the last penetrated plies of each panel. A mountain-shaped distribution of delamination was noticed in Figure 10 for both conventional TTAI reinforced panels P10B and P17C, with the peaks located right after the last penetrated reinforcement ply, which is also considered as the boundary between Section B and Section C. 

The energy absorption should be considered to be more of a dynamic process, which takes into account the delamination introduced both and after the fibre fracture. The delamination damage obtained from the post-mortem examination of the non-perforated panels included the delamination caused by the bouncing back of the projectile along its penetration path before it finally stops in Section B. Although it is difficult to separate the delamination that resulted before and after the fibre ruptures, an overall smaller delamination volume still indicates that less impact energy was absorbed through delamination damage. This means that the impact energy was dissipated more in other failure modes, including fibre rupture and matrix cracking. 

Apparently, the P10B did better in utilising the fibres for energy absorption, not only because there was a smaller damage volume but also in terms of the number of reinforcement plies penetrated and the smaller thickness increase compared to P17C. Hence, under the same areal density of the composite panels, the use of reinforcements with a greater ply areal density and smaller number of reinforcement plies would result in less damage. It is also worth mentioning that only two types of TTAI reinforcements were compared in the current study. Although the two types of TTAI were designed to have the same weave tightness, the effects of each individual reinforcement structural parameters, different reinforcement orientations within the panel (such as [0/90]_n_ and differently shaped impact projectiles on the ballistic performance of the composites still require further investigation.

## 4. Conclusions

This paper presents an experimental study examining the effects of panel construction on the ballistic performance of multiply 3D TTAI fabric reinforced composites. Two types of composites reinforced by TTAI with different ply areal densities and number of plies were compared for their ballistic damage morphology under the same panel areal density. Non-destructive testing methods of through transmission ultrasonic C-scan and X-ray CT were adopted for the damage evaluation. We found that the P10B reinforced by 10-ply heavier TTAI had better overall ballistic performance than that of the P17C. The penetration percentage, indentation, planar distribution and volume of delamination of P10B were all smaller than P17C. All these findings indicate that the use of reinforcements with a greater ply areal density and smaller number of reinforcement plies would result in less damage. Three distinctive damage sections through the thickness of the impacted panels were noticed. The differences in failure modes within different sections is important for the design of multiply fabric reinforced hybrid ballistic composites accordingly. With TTAI fabrics being highly mouldable over doubly-curved parts, the study presented would guide the design of multiply TTAI continuously reinforced composites for ballistic protection.

## Figures and Tables

**Table 1:** Weave specifications of the composite reinforcements.

Index	Yarn	Fabric				
	Linear Density (tex)	Weave Structure	Ply Areal Density (g/m^2^)	Number of Weft Layers	Thread Density (thread/cm)	
					Warp Density (ends/cm)	Weft Density (picks/cm)
B	168	Conventional TTAI	618.67	5	10	28
C	93	Conventional TTAI	382.92	4	11.5	30

**Table 2:** Specifications of the designed composite panels.

Index	Reinforcement Type	Number of Fabric Plies	Areal Density (kg/m^2^)	Fibre Volume Fraction (%)	Thickness (mm)
P10B	B	10	11.95	54.15	8.89
P17C	C	17	12.02	56.37	8.91

**Table 3:** Mechanical properties of the reinforcing Twaron yarn and cured epoxy resin.

Materials	Density (g/cm^3^)	Young’s Modulus (GPa)	Tensile Strength (GPa)	Elongation at Break (%)
Twaron^®^	1.44	60–80	2.40–3.60	3.00–4.40
Epoxy	1.25	2.86–3.00	70–74	4.60–5.00

**Table 4:** Imaging parameters of the X-ray CT scans.

Accelerating Voltage (kV)	Filament Current (μA)	Filter	Exposure Time (ms)	No. of Projections	Voxel Size (μm)
225	135	1 mm Tin	708	3142	59.83

**Table 5:** Ballistic performance of the non-perforated composites.

Specimen Type	Impact Energy (J)	Planar Damage Area (cm^2^)	3D Damage Volume (mm^3^)
P10B	115.10	49.78	753.72
P17C	113.84	74.96	1975.69

**Table 6:** Analyses on the impacted 2D slice images.

Specimen Type	Projectile Angle (°)	Number of Penetrated Plies	Penetration Depth (mm)	Maximum Thickness (mm)	Penetrated Percentage (%)	Thickness Increase Ratio (%)
P10B	0.00	6	7.35	11.89	61.8	33.8
P17C	−12.64	12	9.31	12.71	73.3	42.7

**Table 7:** Dimensions of the damaged areas of P10B and P17C.

Specimen Type	Length of Long Axis (cm)	Length of Short Axis (cm)
P10B	8.42	4.98
P17C	10.85	5.62

**Table 8:** Mechanical properties of single-ply conventional TTAI reinforced composite P1C.

**Young’s Modulus (GPa)**	Warp direction	13.40
	Weft direction	60.05
**Tensile strength (MPa)**	Warp direction	222.10
	Weft direction	519.99
